# Correcting Decalibration of Stereo Cameras in Self-Driving Vehicles

**DOI:** 10.3390/s20113241

**Published:** 2020-06-07

**Authors:** Jon Muhovič, Janez Perš

**Affiliations:** Faculty of Electrical Engineering, University of Ljubljana, Tržaška Cesta 25, SI-1000 Ljubljana, Slovenia; janez.pers@fe.uni-lj.si

**Keywords:** visual stereo, recalibration, optimization, ranging

## Abstract

Camera systems in autonomous vehicles are subject to various sources of anticipated and unanticipated mechanical stress (vibration, rough handling, collisions) in real-world conditions. Even moderate changes in camera geometry due to mechanical stress decalibrate multi-camera systems and corrupt downstream applications like depth perception. We propose an on-the-fly stereo recalibration method applicable in real-world autonomous vehicles. The method is comprised of two parts. First, in optimization step, external camera parameters are optimized with the goal to maximise the amount of recovered depth pixels. In the second step, external sensor is used to adjust the scaling of the optimized camera model. The method is lightweight and fast enough to run in parallel with stereo estimation, thus allowing an on-the-fly recalibration. Our extensive experimental analysis shows that our method achieves stereo reconstruction better or on par with manual calibration. If our method is used on a sequence of images, the quality of calibration can be improved even further.

## 1. Introduction

Stereo systems are widely used for autonomous vehicle navigation as they give a dense 3D reconstruction of the area in front of the cameras and include depth information that is invaluable for obstacle detection and avoidance. Due to movement, temperature changes and vibrations during operation, the physical relationship of the cameras in a stereo system can change. In that case, the calibration no longer adequately describes the system properties. Such a system starts to perform poorly and recalibration is needed. If the stereo system is one of the primary navigation tools for an autonomous vehicle, undetected decalibration is extremely dangerous as it may cause the vehicle to go “blind”.

The first calibration is performed at assembly, but the movement, temperature changes and handling, even in storage, can cause decalibration at a later time. In vehicles that move over rough terrain or have other sources of vibration (e.g., heavy-duty internal combustion engines in trucks, tractors and boats), decalibration of extrinsic parameters can be a regular occurrence, especially given the constraints placed upon the design of a stereo rig. While intrinsic parameters are enclosed in each camera-lens system and can usually be effectively proofed against accidental decalibration, this does not necessarily hold for extrinsic parameters which occur regularly in practice.

Practical problems in calibration arise because of the following real-world constraints:

- The real-world stereo system that benefits self-driving vehicles is usually scaled up significantly to provide a useful range. That means a much wider baseline and consequently a higher sensitivity to mechanical stress.
- Sensors in self-driving vehicles are subject to size, weight and price constraints. It is not possible to use a heavy, sturdy construction in all cases. For example, the perception system that we use in the experiments has been mandated by constraints of the autonomous vehicle, which in this case is a small USV and requires a low center of gravity.

**Figure 1:**
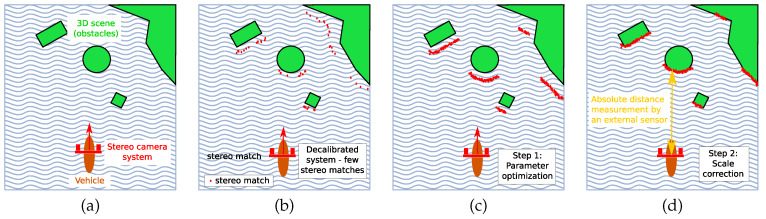
Our approach, from left to right. (**a**) A vehicle observes its surroundings using stereo camera system. (**b**) Decalibration results in failure of the stereo method. (**c**) Camera parameters are optimized to maximize amount of stereo matches. (**d**) External sensor is used to complete the process.

**Figure 2:**
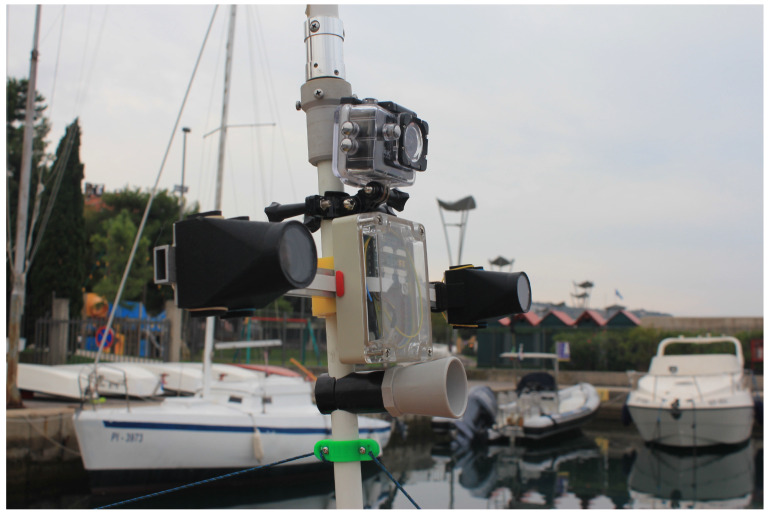
Our stereo system mounted on the USV.

During our work with USVs we have observed several occurrences of decalibration which were revealed only after inspection of captured data. In particular, decalibration due to mechanical stress results in the following:

- Loss of depth estimation coverage. The amount of the recovered depth pixels drops, as illustrated in Figure 1b). The reason for this is as follows. In theory, any correspondence between two points in the left and right image plane can be used to derive depth in a calibrated stereo system. This approach relies on highly distinctive image points (keypoints, e.g., SIFT keypoints [1]), which are matched between the left and the right image, with only a few constraints. The requirement that points are highly distinctive leads to the concept of *sparse stereo reconstruction*, where depth can be reliably established, if only for a few hundred to few thousands of points in an image pair. However, widely used stereo algorithms (which are the focus of our research) often aim to provide *a depth image*, which is *a dense stereo reconstruction*, where, if possible, each pixel in the image is assigned its depth. Dense stereo algorithms are preferred in applications that rely on point cloud processing, such as 3D obstacle detection from stereo [2]. These algorithms are highly optimized for real-time use, and employ several optimizations to speed up processing. First, the geometrical relations between the cameras, obtained during the calibration are used to rectify and align images, so that epipolar lines are horizontal. Therefore, such stereo algorithms restrict the search for stereo matches along the rows of pixels, which is highly efficient in a computational sense. Second, to prevent combinatorial explosion, only a small, viable range of pixels is tested for a potential match. That range is one of the parameters of the stereo algorithm and has to be adjusted for desired performance, depending on the application and scene geometry (e.g., expected range of depths the system should observe). Due to these optimizations, even a slight decalibration results in breakdown of algorithm performance, visible as a reduction in a number of viable depth pixels returned.
- Loss of accuracy. Loss of accuracy is almost impossible to detect during the actual mission if vehicle is equipped only with stereo system to measure distance to obstacles. This is often the case with lightweight and low cost systems such as the one used in our experiments (Figure 2), as a pair of stereo cameras can be significantly less expensive than a full-fledged, multi-channel LIDAR).

The dangers of undetected loss of accuracy are obvious. However, loss of depth estimation coverage (the reduced amount of pixels for which the algorithm even provides the depth) puts the vehicle into an extremely dangerous condition where obstacles may not be detected at all.

**Figure 3:**
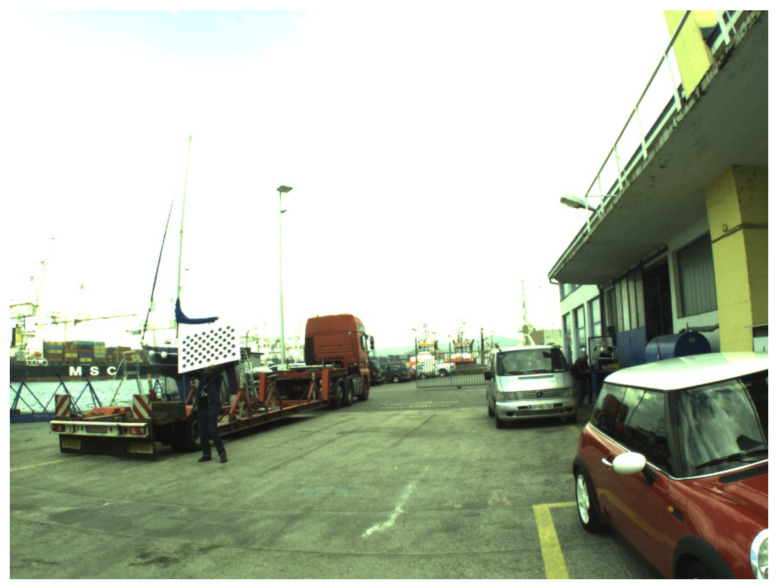
Calibration of a stereo system that is mounted on an unmanned surface vehicle (USV) is a completely different endeavour than calibrating the stereo rig in the laboratory. Note that “in the wild” it is practically impossible to cover the whole image area with the calibration target, unless one of the operators is able to climb while holding the target.

In addition to the problem of decalibration during the autonomous vehicle mission, there is also danger of stereo system decalibration during handling and transport of the vehicle. Full manual calibration before each USV mission, as shown in Figure 3 is time consuming and might not be possible due to lighting conditions, limited battery life, weather, or time constraints. In vehicles operated by users without engineering knowledge, the full calibration procedure including target movement is outright impossible.

The accuracy of stereo 3D scene reconstruction profoundly relies on the accuracy of calibration. In the context of stereo vision, calibration of the system’s extrinsic parameters is a process where a target of precisely known dimensions is observed by both cameras, and a number of parameters that define the transformation from world coordinates (X,Y,Z) to image coordinates in left $\left(u_{l},v_{l}\right)$ and right camera $\left(u_{r},v_{r}\right)$ is calculated. During the calibration process, the intrinsic parameters of each camera and the physical relationship between the cameras (i.e., the extrinsic parameters) have to be calculated. In the paper we will refer to this calibration as “manual”, even though it is fairly automatic, but still requires some level of human involvement. The most tedious task in manual calibration is changing the location of the target so that it covers most of the area where we want to perform 3D reconstruction. In self-driving vehicles that may be huge (tens of meters in front of the vehicle, and 10–30 m in width).

The usual assumption in stereo systems is that the cameras have the same orientation, and are displaced in exactly one dimension (the displacement between cameras is known as “baseline distance”). Usually the displacement is on the *x* axis, which allows stereo matching algorithms to exploit the properties of epipolar geometry. If the system suffers minute changes to its physical configuration, the epipolar constraints are violated and the assumptions of stereo matching algorithms no longer hold.

As a manual calibration is not always feasible, a method to detect and possibly correct decalibration is needed. Such an automatic method could significantly simplify the deployment of mobile robots and increase safety of self-driving vehicles, and driver-assistance systems. Even a poor, but working calibration would be a better solution than a system that is blind and non-functional. A direct calibration using feature points and the essential matrix is theoretically possible, but due the highly varying environments autonomous vehicles operate in, it might not be viable or reliable.

While it is not likely that we could achieve a very precise calibration solely using our cost function optimization, a good use case for our approach is recovery from decalibration in action. If decalibration occurs during a mission, our USV might be completely unable to detect nearby obstacles and calculate its relation to them. In that case even a relatively rough stereo scene reconstruction can be of great use to finish the mission and return to port. Another use case is to make retroactively useful the data recorded while the system was decalibrated. If we record stereo image pairs from a system with unknown calibration, the stereo data cannot be recovered if manual calibration was not performed immediately after the mission’s end.

Our main contribution is thus a new on-the-fly recalibration method for stereo systems that allows recalibration in real time. The method is based on directly estimating the quality of stereo reconstruction using our novel cost function. Recalibration of the system is then performed by optimizing over the cost function, and scale is obtained using single measurement from inexpensive range sensor. A larger number of images can be used to even further improve the calibration as they are captured during a mission. The method is also fast enough to allow real time calibration correction.

## 2. Related Work

Geometry of a stereo calibration is a well-researched problem. An additional push into research on stereo happened several years ago with realistic prospects of driver assistance and obstacle detection systems, with the ultimate goal of developing fully self-driven vehicles. The base for stereo vision methods was established as early as 1981 by Longuet-Higgins [3], which describes the widely known 8-point algorithm for reconstructing a 3D scene from point matches in images. Later works, such as Hartley’s [4] and Nistér’s [5] expand upon Longuet-Higgins’ work and present improvements on the solutions of the pose reconstruction problem.

Significant research has been invested in automatic camera and stereo system calibration and strong baseline methods have been established [6,7]. These set up the standard approach of matching the detected features in camera images to the patterns that are known upfront and thus producing a system of linear equations that can be solved with a closed form solution. Similarly, non-linear lens distortion parameters can be estimated. The work of Takahashi et al. [8] deals with estimating the rotation parameters of a stereo system given the observed points which are calculated via boundary representations of input images. The works by Dang et al. [9,10,11] explore the calibration of a stereo system with movable cameras by using bundle adjustment on tracked points in sequential images combined with epipolar constraints. The calibration is then continuously optimized using an Iterated Extended Kalman Filter (IEKF). A similar approach combined with CNN image segmentation was presented in [12,13]. The work by Collado et al. [14] deals with determining the height, roll and pitch of a stereo system used for driver assistance systems. Here, the road lanes detected in the images captured from a car mounted stereo system are used as a calibration pattern under assumptions on planarity and parallelism of the lanes on a straight road segment. The research by de Paula et al. [15] also exploits known features on the road to achieve on-the-fly calibration of a stereo system. Methods directly estimating stereo parameters from image contents via visual landmarks were also researched [16,17].

Research into self-driving technology introduced concepts that have not been popular before, such as systems with heterogeneous cameras. Such systems need adapted calibration targets and different methodology for efficient (practical) calibration, as for example [18]. The introduction of high definition cameras and related inexpensive hardware had a similar effect, which called for efficient calibration of high-definition stereo rigs [19]. The consequence of stereo imaging systems entering widespread use is indeed the search for methods that are easy to operate and still provide high-quality stereo calibration, one of such methods is presented in work by Sun et al. [20]. Interesting common point with our system is that they specifically target large calibration volumes, which cannot be efficiently calibrated by the conventional checkerboard pattern methods. The acknowledgement that fiducial marker-based (checkerboard) approaches to calibrate multiple cameras are impractical in the field of self-driving vehicles, is the reliance on natural features through the SLAM based approaches, as in [21]. The motivation for our approach is somewhat similar.

Several works were also published on the topic of calibrating stereo systems for USVs [22,23,24,25,26]. Their approaches range from exploiting features such as horizon estimation to calibrate a wide-baseline stereo system to using the water surface as a reference for calibration or employing deep learning to extract and match features from image data.

Finally, some existing methods use additional sensors to complement the camera system or even seek to calibrate sensors with respect to the camera [27]. In [28], authors describe a system which calibrates the stereo and LIDAR without the use of calibration targets, relying only on the observed surroundings of the vehicle.

Our approach to performing adjustments to calibration is largely introspective, which means that it does not need full ground truth from additional sensors—it performs optimization on acquired image data only, after which only one measurement from inexpensive distance sensor is needed to establish the scale. It can be argued that it is simple and self-evident, but to best of our knowledge, the method has not been published before. The closest to our idea is in mentioned a paper by Konolige [29], where the author describes two measures of the quality of stereo calibration and proposes a hierarchical search strategy to achieve automatic calibration, but the method is not described in much detail.

## 3. A Method for Unsupervised Stereo Recalibration

In this section we outline the theoretical background for our method. Section 3.1 describes our stereo cost function, while Section 3.2 details how the cost function can be used in calibration optimization. Finally, Section 3.3 addresses the problem of absolute scale when optimizing extrinsic parameters using the cost function.

The key idea of our method is the observed fact that violation of epipolar constraints as a consequence of decalibration inevitably causes stereo algorithms to detect fewer matches. They are optimized to search for matches along epipolar lines only, and the greater the decalibration, the fewer matches will be detected. It should be noted that by matches we mean the matches within a block matching stereo algorithm, not the more general feature matches (e.g., SIFT, [1]). Our observation is that the number of pixels recovered by a stereo matching algorithm is an indirect measurement of the calibration quality. Our goal is to design a cost function that reflects the quality of extrinsic calibration. Such a function can then be used to track the quality of calibration during optimization and thus a higher quality calibration may be obtained.

### 3.1. Cost Function

Calibration of intrinsic camera parameters is required for any stereo algorithm. We assume calibration of each camera was performed and calibration matrices $C_{1}$ and $C_{2}$ are available. Aside from intrinsic parameters, the output of any stereo matching algorithm also depends on the input images $I_{l}$ and $I_{r}$ and stereo system extrinsic parameters. These are usually defined as a rotation matrix *R* and a translation vector *T*. The rotation matrix *R* can be represented as Euler angles that describe the rotation about each of the three axes, namely roll, pitch and yaw (ϑ, φ, ψ, respectively). Let Ω be a vector of extrinsic parameters of the stereo system.

(1) Ω=[ϑ,φ,ψ,x,y,z].

We define our cost function $f:R^{6}\to \left[0,1\right]$ as the ratio between the calculated disparity points and the theoretical maximum of disparity points. The cost function is therefore

(2) $$f\left(\Omega \right)=\frac{\sum_{x\in d(\Omega )}\min\left(1,x\right)}{N}$$

where d(·) is the stereo matching function that produces the disparity image and *N* is the number of pixels in the disparity image. Both camera matrices and input images are omitted for brevity as they are not the focus of our function evaluation and they remain fixed when comparing different calibration parameters. The value of our cost function when given some set of extrinsic parameters will be referred to as stereo score from here on. If we assumed a good calibration, the cost function value would depend solely on the scene depicted in the input image pair, but when calibration is compromised this adversely affects the stereo score. If we fix the input image pair we are able to use the stereo score to improve the calibration. This is performed by finding

(3) $$\Omega_{opt}=\underset{\Omega \in R^{5}}{arg max}f\left(\Omega \right),$$

i.e., some set of extrinsic parameters that maximizes our cost function while keeping the other parameters fixed. While generally 3D pose has 6 degrees of freedom, we fix the offset along *x* axis to our system baseline (which is known) because it does not affect the stereo quality, but only the absolute scale.

The evaluation of our cost function relies on using a stereo matching function d(·) to obtain a disparity image. A fast and reliable stereo method is needed in order to facilitate the usage on an autonomous vehicle. In our setup we use stereo block matching [29], which is not only fast but can also be finely tuned to work under maritime visual conditions. More generally, any stereo method that uses epipolar constraints to reduce the stereo matching search space only to horizontal lines should work with our cost function.

### 3.2. Optimization Methods

In the field of camera parameter optimization, standard optimization techniques such as Levenberg-Marquardt method are often applied, but they require analytical derivatives. Since the function f(·) cannot be derived analytically, numerical derivation has to be applied. This prompts the usage of a standard gradient descent optimization approach that proved to be more robust in this use case than some well established techniques. We use numerical derivation of our cost function which requires 2n evaluations of the function for each of the *n* parameters. The optimization additionally depends on two other parameters: δ specifies the offset used to calculate the numerical derivatives and γ which is the step size used to move in the direction of the calculated gradient. Additionally, backtracking line search is included in the optimization to adjust the step size γ if the current step size would cause a reduction in the objective function. This is included to avoid oscillations near the maximum and to ensure convergence.

### 3.3. Scale Correction

Our method intentionally avoids optimizing the baseline because it has no bearing on the *shape* of the stereo scene reconstruction. However, perturbations of other extrinsic parameters can have twofold effects: on one hand they can improve the scene reconstruction (i.e., stereo score) which is desirable. On the other hand the absolute scale that in a perfectly calibrated stereo system depends only on the baseline (since the focal length is fixed) can get corrupted with changes in other extrinsic parameters. In order to fully recover both the scene shape and the absolute scale without manual calibration, some sort of absolute distance measurement is required. If the absolute depth for some pixel in the camera image can be measured, we can calculate the appropriate scaling factor that scales the depths calculated by the stereo system to the correct absolute scale.

## 4. Experiments

Our method was extensively tested on a real USV. In the period of working on the USV that is equipped with stereo camera system (spanning 7 years), manual stereo calibration using calibration targets was performed 11 times. Usually the system was manually recalibrated whenever the stereo method’s performance noticeably degraded. We observed that the differences between them pre- and post-calibration could reach as high as 2.5${}^{\circ }$ in rotation and up to 8 mm in translation. This shows that decalibration both during the vehicle operation and due to storage can be significant. We also have image data from 39 USV sessions available and are thus able to retroactively check the calibration quality. The experiments in this section were all performed on image pairs from 16 different USV missions. The missions were chosen by their proximity to manual calibrations, so the effect of decalibration can be well observed. Image pairs were uniformly sampled from the chosen missions and manually filtered so the contents are as varied as possible. A total of 300 image pairs were used in the experiments. The link to the publicly available dataset data is included in the Supplementary Materials.

### 4.1. Detecting Decalibration

In order to evaluate whether our cost function correctly reflects the calibration quality we need to establish that known poor calibration on average produces lower scores than high quality calibration. For this we used our collection of manual calibrations and images captured on USV missions. We can assume that manual calibrations were performed only after degradation of stereo algorithms was observed. Thus by evaluating our cost function using a calibration that performed poorly and corresponding camera images, we expect a relatively low stereo score. If we additionally assume that only manual calibration was performed after a mission with poor stereo performance (instead of a complete refitting of the stereo system) using the subsequent manual calibration should produce higher stereo scores.

The images were uniformly sampled from 16 missions and our cost function was evaluated on all of them using both old and new calibration parameters. Table 1 shows the mean stereo scores for each sequence. In most cases the later calibration produced higher values of our cost function. This shows that our cost function correctly reflects the quality of calibration which was improved after performing manual calibration.

It should be noted that occasionally an old calibration might produce larger stereo scores. As mentioned earlier, this might be due to complete refitting of the stereo system. This would change the physical configuration so that the new calibration would not correspond well with images previously captured.

### 4.2. Stereo Cost Function Calibration Sensitivity

**Figure 4:**
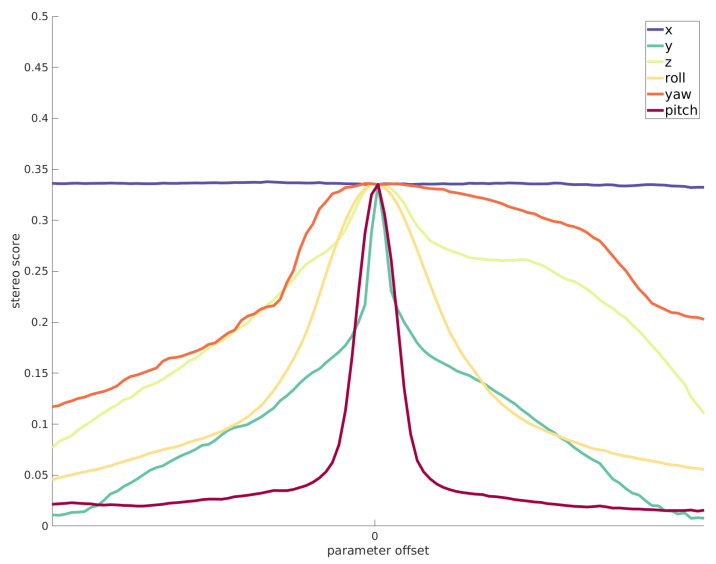
The effect of parameter noise to the stereo score function.

To analyze the behavior of our cost function from Section 3.1 we added noise to each of the extrinsic parameters of the stereo system and observed the stereo score. Figure 4 shows the stereo score with relation to changes in parameters. We can see that parameter *x* has little to no impact on the disparity shape and therefore also not on the stereo score. This is predictable as the stereo baseline only determines the calculated distance to the scene but not its shape. A similar thing holds for the yaw angle ψ, which significantly changes the calculated disparity values but does not significantly impact the shape reconstruction ability of the stereo algorithm. The opposite holds for pitch angle and *y* axis which are the most sensitive calibration parameters, because changes to them directly violate the epipolar geometry assumptions of a stereo system.

### 4.3. Calibration Optimization Analysis

In Experiment Section 4.1 we showed that our stereo score correlates highly with the quality of stereo calibration. In Experiment Section 4.2 we showed that adding noise to extrinsic parameters of the system can severely reduce the stereo score of a manual calibration. But if the manual calibration is no longer correct because of decalibration, slightly changing calibration parameters could result in a higher stereo score and more information about the scene can be recovered. In this experiment we used our method to improve the stereo scores of sequences from Experiment Section 4.1. For the initial calibration, the calibration available at the time of the mission was used.

**Figure 5:**
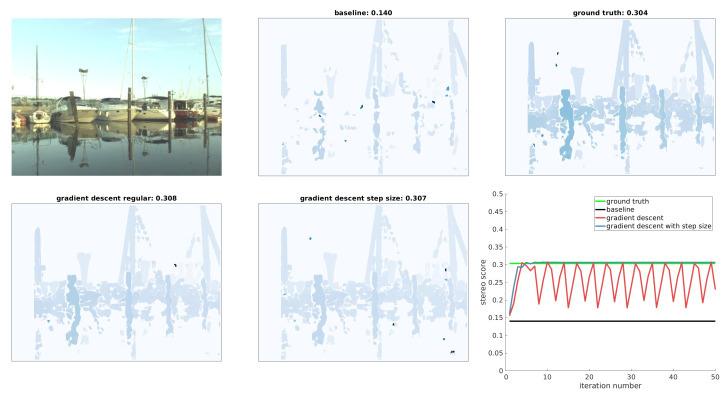
Disparities before and after optimization with regular gradient descent and with gradient descent using step size adjustment. In the bottom right is the graph of stereo scores during the optimization.

In Table 2 we show the average stereo scores before and after correction. The results are presented per sequence, since the initial calibrations and visual characteristics change drastically between sequences. It can be observed that our optimization method always improves the stereo score with regard to the initial calibration. The relative improvement depends on the severity of decalibration. Our method’s optimization converges in around 7 iterations on average, which makes it fast enough for real time calibration correction. Figure 5 shows the disparities before and after optimization as well as the graph of the stereo score during optimization. Both regular gradient descent and gradient descent with backtracking line search. While regular gradient descent achieves high scores, its performance depends highly on the fine tuning of parameters, where too small γ can prevent convergence and too large γ can cause oscillations. Backtracking line search solves both of these problems and makes the optimization method work on all tested images without changing the method parameters. It can be observed that the optimization approach achieves near ground truth quality both visually and with regard to stereo score and a very large improvement relative to the initial calibration is clearly seen.

### 4.4. Fully Automatic Calibration

In previous experiments we showed that our method can improve stereo scores on a variety of different image pairs. Given the fact that physical configurations of the stereo system can vary by a large amount over time, we also wanted to test initial conditions with no bias whatsoever. We called this ‘zero calibration’ and here the initial condition of the system is characterized only by the baseline, other translational and rotational parameters are assumed to be zero. This removes the need for a previous calibration and serves as a neutral starting point for the optimization. We performed both calibration correction and zero calibration on all available image pairs. Table 3 shows that correcting an existing calibration yields the best results most of the time, while using no a priori information often produces results that are often near as good. A good amount of time, the manual calibration results are better than either optimization, but as previously stated, the calibration correction is not meant to replace manual calibration, but only recover as much of stereo functionality as possible.

In general, the speed of our method is bounded by the speed of the block matching algorithm. In our experiments we used the OpenCV implementation of the stereo block matching algorithm which takes on average 0.025 s to complete on an Intel Core i7-8700k CPU. In each iteration of our method’s optimization, the full stereo matching pipeline has to be run twice for each of the 5 parameters. This means that a single iteration of our optimization should take around 0.25 s. The average number of iterations for all our test image pairs is 9.08±5.97, so the full optimization is expected to finish in around 2 s.

### 4.5. Sequential Calibration

Experiment Section 4.4 shows that stereo score can be improved with optimization. But this does not mean that an optimal point was reached that negates the physical change in the stereo system that caused the low stereo scores in the first place. This experiment serves to show that our optimization approach generally converges towards the same solution regardless of the image pair used (all image pairs must have been captured on the same mission). Not only that, we can also show that sequentially performing the optimization on different image pairs improves the calibration quality more than performing it on a single image pair. This is a good starting point for a system for online calibration correction, because a lot of image pairs can be collected during a mission, and their joint information can produce higher quality results.

**Figure 6:**
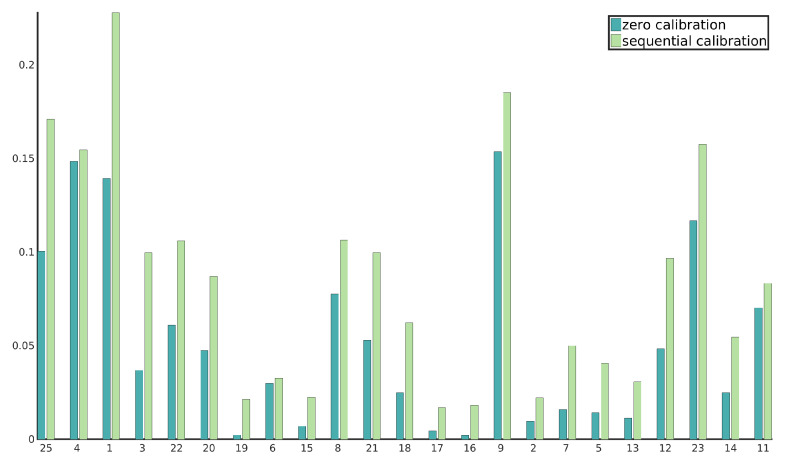
Difference in zero calibration and sequential calibration scores for one of the permutations of images from sequence 82.

To support our claim, we performed 5 runs of sequential calibration on each of the selected sequences. A random permutation of image pairs was generated for each run. For each image pair, we once performed a full calibration with no a priori information. At the same time, we separately improved the calibration we obtained from the previous image pair optimization. Table 4 shows that the mean score for a sequence does not vary much with respect to the sequence ordering as well as that generally higher stereo scores are obtained when sequentially correcting the calibration using many different image pairs. Figure 6 shows the stereo scores for each image pair in a sequence for both zero calibration and sequential calibration. It can be observed that stereo scores for the sequentially corrected calibration are consistently higher than per-image-pair zero calibration scores.

### 4.6. Stereo Score Potential Based on Image Content

When calculating the stereo scores for different image pairs from the same mission, it can easily be seen that their scores vary greatly (c.f. Figure 6). The stereo score depends on the presence of uniquely textured regions and their distance to the stereo system. This is dependent on the image content as some images from a mission obtain relatively low stereo scores even with a known good calibration. It is beneficial to use images with a large potential for stereo matches to perform calibration optimization. If images do not contain sufficiently textured regions, no block matching stereo algorithm can perform well. It is also possible (for low visibility cases) that there are almost no disparity points returned (i.e., the stereo score is near zero). In that case, the neighborhood in the optimization search space might not be descriptive enough for an optimization method to gain traction.

**Figure 7:**
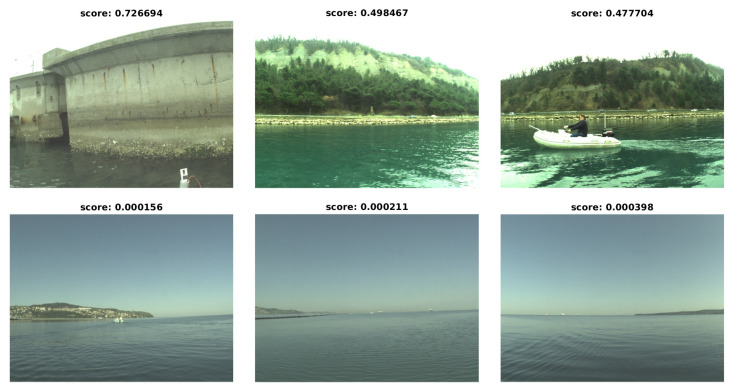
Images with highest and lowest stereo scores when using a known good calibration. Top row shows images with highest scores, while the bottom row depicts images with lowest scores.

Figure 7 shows images with the highest and the lowest stereo scores. The analysis of different image contents gives us a good rule for manual selection of image pairs on which optimization is plausible, and could also serve as a base for automatic image pair selection if automatic online calibration correction is performed (i.e., during a mission the system only performs calibration optimization using scenes with enough textured regions). USV heading information coupled with a map, or image content analysis, such as semantic segmentation [30,31] could be used to choose good images for optimization.

### 4.7. Enforcing Scale

Our method can be used to obtain a useful scene reconstruction from a decalibrated system. While that is sufficient for obstacle detection and object tracking, obstacle avoidance applications might require a more precise estimate of the distance. Since our method is based on reconstructing the scene and not perfect calibration, errors in scale estimation can occur (c.f. slight color differences in ground truth and optimized disparity images in Figure 5) in addition to any errors in scale that were already present after decalibration event. While the stereo baseline is fixed during the optimization, changes in absolute scale can still be observed due to the changes in yaw angle, for instance.

To address this problem, an external measurement is needed. We define “external” as related to the stereo system—e.g., having laser range finder measurement as an ultimate ground truth. We calculate the per-pixel absolute error between the external depth measurement, and the corresponding area of pixels in our estimated depth map and average it to obtain the scaling factor by which the estimated depths are rectified to improve the depth accuracy at all estimated pixels.

For the purpose of this experiment, we used two sets of our archived stereo system data, where the USV was, by chance, observing the same scene during two missions. During the acquisition of one of those sets, the system was decalibrated, during the acquisition of the the other, the system was freshly calibrated after a full calibration onshore (this was our reference set, which was used in place of external sensor, purely for the purpose of this experiment).

We selected 40 image pairs from both sets where the reference and decalibrated depth images are well aligned. While both the reference and our method’s results achieve good stereo scores and high quality of stereo scene reconstruction, they are not always aligned. The selected depth images have the property of being aligned, therefore all image objects lie at the same coordinates in both compared images. Aside from some difference in density of reconstruction, the only difference between the depth images is in depth values. To simulate a sparse external sensor like LIDAR, radar, or single beam laser ranger, we only keep 0.1% of points from the reference depth images produced by manual calibration. From these image pairs, we estimated the mean absolute error of the depth produced by our automatic calibration is 2.4168±2.8716. After we correct the relative depth using the correction factor from the reference calibration, the mean absolute error drops to 0.9182±0.7464 which is low enough for safe navigation, or at least safe return to port, where the USV can be properly serviced.

In practice, even inexpensive absolute distance sensor (e.g., radar or a single point laser range sensor) could be used to provide the depth information. Since the correction factor should be constant for all depths, a single absolute depth measurement should be enough for depth correction, but more measurements, such as observing single beam laser range sensor through sequence of images would increase the robustness of such correction. The prerequisite is that the external geometrical relation between stereo system and the distance sensor was not heavily affected by decalibration event.

## 5. Conclusions

We proposed a new method for automatic recalibration of a decalibrated stereo system on an autonomous vehicle. A cost function was formulated that reflects the quality of extrinsic stereo parameters with regard to stereo block matching results. The cost function is based on the ratio of valid disparity points to theoretical disparity point maximum from a stereo matching algorithm and it is a good metric over which we perform optimization. A long history of work with stereo-based USV navigation provided us with a historical record of calibrations and corresponding image sequences, which enabled us to truthfully simulate real world conditions. We demonstrated that even though decalibration during vehicle operation can occur, our method achieves results that consistently rival the quality of the results produced by a manual calibration. Additionally, the results of our method can at times exceed the initial score of the manual calibration, thereby improving the quality of the disparity image even further. Additional value of our method can be gained from the fact that we can use it to retroactively calibrate the camera for past USV missions. If the stereo system was not properly calibrated before a mission (or decalibration occurred during operation), the stereo data could be useless. By applying our method, we can calibrate the system for data that was acquired with a poorly calibrated system. Given the speed of our method, it can also be used to correct the calibration during vehicle operation in real time. Additionally we showed that an even better calibration estimate can be obtained when using several image pairs sequentially.

Given that correct absolute scale can be lost during optimization, we also proposed and tested a correcting method using absolute distance measurements from an external sensor. This can be performed with as little as one measurement point from a calibrated absolute distance sensor (e.g., laser). The most obvious problem here is the decalibration of the distance sensor in relation to the stereo camera system because of the mechanical stress. By mounting the distance sensor close to the coordinate system of the stereo camera system, ideally in the middle of the structure connecting both cameras, this problem can be minimized (and collocation of range and stereo measurements slightly simplified). In such a setup, collocation could be done by using the distance sensor which emits the (laser) light in near-infrared range, using visible-light blocking filters on cameras for the purpose of initial factory calibration and removing them for actual deployment. It should also be noted that our proposed calibration method in two steps actually offers a degree of robustness against decalibration of the distance sensor: since the shape of the 3D scene is established before the scaling, the vehicle could conservatively seek for appropriate object to do the self-calibration of scale (e.g., a sufficiently large flat area, which can be detected as soon as optimization step is finished). The larger the flat area, the larger could be angular deviation of the range sensor from the factory preset point, to still provide decent calibration of scale. We consider this a possible future work.

Given the robustness of our method to initial parameters, it could potentially be used instead of a manual calibration or at least significantly reduce the frequency of manual calibration. Further work will be focused in the direction of possible joint calibration of multiple sensors (stereo system + LIDAR). A similar procedure could also be used to detect decalibration of intrinsic camera parameters that might be prone to decalibration (most notably the center of the image plane, and focal length).

## Figures and Tables

**Table 1:** Mean stereo scores for different manual calibrations.

Sequence	$\mu_{\mathrm{old}}$	$\mu_{\mathrm{new}}$
60	0.196	0.064
64	0.006	0.153
65	0.004	0.147
67	0.093	0.01
71	0.003	0.109
75	0.15	0.169
82	0.06	0.063
83	0.131	0.137
90	0.155	0.128
96	0.224	0.331
99	0.021	0.098
100	0.027	0.091
101	0.033	0.054
102	0.006	0.052
103	0.037	0.167
104	0.061	0.184

**Table 2:** Comparison of stereo scores after correction with initial stereo scores. We show the initial scores in the first column ($\mu_{old}$), column $\mu_{c}$ then shows mean stereo scores for the corrected calibration. The highest value in a row is denoted in bold.

Sequence	$\mu_{\mathrm{old}}$	$\mu_{c}$
60	0.196	**0.239**
64	0.006	**0.151**
65	0.004	**0.173**
67	0.093	**0.095**
71	0.006	**0.147**
75	0.15	**0.195**
82	0.06	**0.078**
83	0.131	**0.152**
90	0.155	**0.189**
96	0.224	**0.275**
99	0.021	**0.148**
100	0.027	**0.159**
101	0.033	**0.131**
102	0.006	**0.105**
103	0.037	**0.188**
104	0.061	**0.214**

**Table 3:** Stereo scores before and after calibration. Columns contain stereo score of previous ($f_{old}$), next ($f_{new}$) and zero ($f_{0}$) calibrations. Last two columns show stereo scores after calibration: $f_{c}$ contains corrected calibration score and $f_{z}$ shows stereo scores obtained starting with zero calibration. The highest value in a row is denoted in bold.

Sequence	Name	$f_{\mathrm{old}}$	$f_{\mathrm{new}}$	$f_{0}$	$f_{c}$	$f_{z}$
60	00000850	0.215	0.102	0.135	0.272	**0.273**
60	00002180	0.066	0.006	0.021	**0.067**	0.067
60	00005950	0.106	0.009	0.037	**0.148**	0.145
64	00008501	0.001	**0.185**	0.008	0.114	0.118
64	00013501	0.014	0.01	0.012	**0.015**	**0.015**
65	00001603	0.014	0.091	0.022	**0.157**	0.153
67	00041065	**0.108**	0.002	0.082	0.105	0.101
71	00001717	0.002	0.173	0.002	**0.202**	**0.202**
71	00003103	0.003	**0.109**	0.005	0.009	0.006
71	00012000	0.009	**0.161**	0.006	0.154	0.154
75	00001810	0.177	**0.366**	0.003	0.217	0.214
75	00004810	0.026	0.051	0.008	**0.053**	0.053
82	00001601	0.200	**0.227**	0.048	0.22	0.203
82	00009601	0.029	**0.045**	0.001	0.042	0.037
82	00017601	0.054	0.035	0	**0.096**	0.093
83	00008401	0.09	0.033	0	**0.11**	0.001
83	00020401	0.203	0.198	0.03	**0.214**	**0.214**
90	00015010	0.195	0.153	0.005	**0.219**	0.216
90	00065010	0.216	0.16	0.006	**0.226**	0.225
96	00005010	0.192	**0.290**	0.041	0.107	0.051
99	00002601	0.036	**0.15**	0.13	0.149	0.149
99	00005801	0.060	0.161	0.142	**0.162**	**0.162**
99	00007601	0.006	**0.092**	0.075	0.091	0.091
100	00000010	0.063	0.103	0.156	**0.169**	**0.169**
100	00035010	0.001	0.009	**0.071**	0.063	0.058
101	00058510	0.043	0.051	0.025	**0.178**	0.177
102	00010210	0	**0.001**	0	**0.001**	0
102	00027210	0	0.039	0.001	0.197	**0.205**
103	00018200	0.005	0.103	0.006	**0.143**	0.14
104	00009001	0.007	0.025	0.002	**0.106**	**0.106**

**Table 4:** Mean stereo score per sequence for all 5 runs with different permutations of image sequences. Here $\mu_{0}$ rows contain mean zero calibration stereo scores for each image pair, while $\mu_{s}$ shows stereo scores for the case of continuous calibration correction.

Sequence		1	2	3	4	5
60	$\mu_{0}$	0.158	0.149	0.154	0.151	0.15
	$\mu_{s}$	0.206	0.22	0.221	0.216	0.224
64	$\mu_{0}$	0.077	0.081	0.079	0.072	0.072
	$\mu_{s}$	0.153	0.15	0.142	0.074	0.144
65	$\mu_{0}$	0.05	0.082	0.06	0.079	0.082
	$\mu_{s}$	0.098	0.192	0.083	0.132	0.17
67	$\mu_{0}$	0.065	0.062	0.078	0.08	0.086
	$\mu_{s}$	0.073	0.075	0.093	0.078	0.093
71	$\mu_{0}$	0.048	0.048	0.048	0.049	0.049
	$\mu_{s}$	0.147	0.136	0.148	0.147	0.148
75	$\mu_{0}$	0.098	0.102	0.102	0.094	0.099
	$\mu_{s}$	0.197	0.168	0.129	0.185	0.18
82	$\mu_{0}$	0.039	0.04	0.039	0.04	0.039
	$\mu_{s}$	0.08	0.069	0.081	0.081	0.078
83	$\mu_{0}$	0.069	0.072	0.072	0.071	0.066
	$\mu_{s}$	0.143	0.155	0.122	0.099	0.143
90	$\mu_{0}$	0.083	0.087	0.079	0.086	0.089
	$\mu_{s}$	0.167	0.183	0.173	0.178	0.177
96	$\mu_{0}$	0.148	0.132	0.152	0.151	0.121
	$\mu_{s}$	0.306	0.291	0.249	0.305	0.254
99	$\mu_{0}$	0.106	0.105	0.104	0.104	0.106
	$\mu_{s}$	0.116	0.116	0.115	0.114	0.117
100	$\mu_{0}$	0.166	0.175	0.175	0.124	0.172
	$\mu_{s}$	0.172	0.179	0.174	0.129	0.172
101	$\mu_{0}$	0.083	0.067	0.075	0.071	0.075
	$\mu_{s}$	0.139	0.118	0.133	0.114	0.135
102	$\mu_{0}$	0.065	0.05	0.062	0.058	0.063
	$\mu_{s}$	0.075	0.066	0.082	0.077	0.077
103	$\mu_{0}$	0.158	0.156	0.147	0.152	0.171
	$\mu_{s}$	0.181	0.182	0.171	0.176	0.183
104	$\mu_{0}$	0.104	0.125	0.114	0.12	0.128
	$\mu_{s}$	0.193	0.224	0.189	0.188	0.201

## Supplementary Materials

The dataset used in the experiments is available online at https://www.vicos.si/Downloads/stereo.
